# Supplementary material for: HIVprotI: an integrated web based platform for prediction and design of HIV proteins inhibitors
Source: J Cheminform. 2018 Mar 9;10:12. doi: 10.1186/s13321-018-0266-y (PMC5845081; doi:10.1186/s13321-018-0266-y)
Supplement: Supplementary file 2 — Additional file 2. Source code of HIVProtI web server. [file 13321_2018_266_MOESM2_ESM.zip › HIVprotI_Source-code/hivprotI/weka-3-6-6/doc/weka/associations/ItemSet.html]

ItemSet


---


|  |  |  |
| --- | --- | --- |
| **Overview** \| **Package** \| **Class** \| **Tree** \| **Deprecated** \| **Index** \| **Help** \| **Weka's home** | |  |
| **PREV CLASS**   **NEXT CLASS** | **FRAMES**    **NO FRAMES**     **All Classes** |  |
| SUMMARY: NESTED \| FIELD \| CONSTR \| METHOD | DETAIL: FIELD \| CONSTR \| METHOD |  |


---


## weka.associations Class ItemSet

```
java.lang.Object
  weka.associations.ItemSet
```

**All Implemented Interfaces:**: java.io.Serializable, RevisionHandler

**Direct Known Subclasses:**: AprioriItemSet, LabeledItemSet

---

``` public class ItemSet extends java.lang.Object implements java.io.Serializable, RevisionHandler ```

Class for storing a set of items. Item sets are stored in a lexicographic
order, which is determined by the header information of the set of instances
used for generating the set of items. All methods in this class assume that
item sets are stored in lexicographic order.
The class provides the general methods used for item sets in class - and
standard association rule mining.

**Version:**
:   $Revision: 1.13 $

**Author:**
:   Eibe Frank (eibe@cs.waikato.ac.nz)

**See Also:**: Serialized Form

---

**Constructor Summary**

`ItemSet(int totalTrans)`
  
          Constructor

`ItemSet(int[] array)`
  
          Contsructor

`ItemSet(int totalTrans,
int[] array)`
  
          Constructor


| **Method Summary** | |
| --- | --- |
| `boolean` | `containedBy(Instance instance)`             Checks if an instance contains an item set. |
| `int` | `counter()`             Gets the counter |
| `static FastVector` | `deleteItemSets(FastVector itemSets, int minSupport, int maxSupport)`             Deletes all item sets that don't have minimum support. |
| `boolean` | `equals(java.lang.Object itemSet)`             Tests if two item sets are equal. |
| `static java.util.Hashtable` | `getHashtable(FastVector itemSets, int initialSize)`             Return a hashtable filled with the given item sets. |
| `java.lang.String` | `getRevision()`             Returns the revision string. |
| `int` | `hashCode()`             Produces a hash code for a item set. |
| `int` | `itemAt(int k)`             Gest the index of the value of the specified attribute |
| `int[]` | `items()`             Gest the item set as an int array |
| `static FastVector` | `mergeAllItemSets(FastVector itemSets, int size, int totalTrans)`             Merges all item sets in the set of (k-1)-item sets to create the (k)-item sets and updates the counters. |
| `static FastVector` | `pruneItemSets(FastVector toPrune, java.util.Hashtable kMinusOne)`             Prunes a set of (k)-item sets using the given (k-1)-item sets. |
| `static void` | `pruneRules(FastVector[] rules, double minConfidence)`             Prunes a set of rules. |
| `void` | `setCounter(int count)`             Sets the counter |
| `void` | `setItem(int[] items)`             Sets an item sets |
| `void` | `setItemAt(int value, int k)`             Sets the index of an attribute value |
| `static FastVector` | `singletons(Instances instances)`             Converts the header info of the given set of instances into a set of item sets (singletons). |
| `int` | `support()`             Outputs the support for an item set. |
| `java.lang.String` | `toString(Instances instances)`             Returns the contents of an item set as a string. |
| `void` | `upDateCounter(Instance instance)`             Updates counter of item set with respect to given transaction. |
| `static void` | `upDateCounters(FastVector itemSets, Instances instances)`             Updates counters for a set of item sets and a set of instances. |

**Methods inherited from class java.lang.Object**

`getClass, notify, notifyAll, toString, wait, wait, wait`

**Constructor Detail**

### ItemSet

```
public ItemSet(int totalTrans)
```

:   Constructor

    **Parameters:**: `totalTrans` - the total number of transactions in the data

---


### ItemSet

```
public ItemSet(int totalTrans,
               int[] array)
```

:   Constructor

    **Parameters:**: `totalTrans` - the total number of transactions in the data: `array` - the attribute values encoded in an int array

---


### ItemSet

```
public ItemSet(int[] array)
```

:   Contsructor

    **Parameters:**: `array` - the item set represented as an int array


**Method Detail**

### containedBy

```
public boolean containedBy(Instance instance)
```

:   Checks if an instance contains an item set.

    :   **Parameters:**: `instance` - the instance to be tested **Returns:**: true if the given instance contains this item set

---


### deleteItemSets

```
public static FastVector deleteItemSets(FastVector itemSets,
                                        int minSupport,
                                        int maxSupport)
```

:   Deletes all item sets that don't have minimum support.

    :   **Parameters:**: `maxSupport` - the maximum support: `itemSets` - the set of item sets to be pruned: `minSupport` - the minimum number of transactions to be covered **Returns:**: the reduced set of item sets

---


### equals

```
public boolean equals(java.lang.Object itemSet)
```

:   Tests if two item sets are equal.

    :   **Overrides:**: `equals` in class `java.lang.Object`
    :   **Parameters:**: `itemSet` - another item set **Returns:**: true if this item set contains the same items as the given one

---


### getHashtable

```
public static java.util.Hashtable getHashtable(FastVector itemSets,
                                               int initialSize)
```

:   Return a hashtable filled with the given item sets.

    :   **Parameters:**: `itemSets` - the set of item sets to be used for filling the hash table: `initialSize` - the initial size of the hashtable **Returns:**: the generated hashtable

---


### hashCode

```
public int hashCode()
```

:   Produces a hash code for a item set.

    :   **Overrides:**: `hashCode` in class `java.lang.Object`
    :   **Returns:**: a hash code for a set of items

---


### mergeAllItemSets

```
public static FastVector mergeAllItemSets(FastVector itemSets,
                                          int size,
                                          int totalTrans)
```

:   Merges all item sets in the set of (k-1)-item sets
    to create the (k)-item sets and updates the counters.

    :   **Parameters:**: `totalTrans` - thetotal number of transactions: `itemSets` - the set of (k-1)-item sets: `size` - the value of (k-1) **Returns:**: the generated (k)-item sets

---


### pruneItemSets

```
public static FastVector pruneItemSets(FastVector toPrune,
                                       java.util.Hashtable kMinusOne)
```

:   Prunes a set of (k)-item sets using the given (k-1)-item sets.

    :   **Parameters:**: `toPrune` - the set of (k)-item sets to be pruned: `kMinusOne` - the (k-1)-item sets to be used for pruning **Returns:**: the pruned set of item sets

---


### pruneRules

```
public static void pruneRules(FastVector[] rules,
                              double minConfidence)
```

:   Prunes a set of rules.

    :   **Parameters:**: `rules` - a two-dimensional array of lists of item sets. The first list of item sets contains the premises, the second one the consequences.: `minConfidence` - the minimum confidence the rules have to have

---


### singletons

```
public static FastVector singletons(Instances instances)
                             throws java.lang.Exception
```

:   Converts the header info of the given set of instances into a set
    of item sets (singletons). The ordering of values in the header file
    determines the lexicographic order.

    :   **Parameters:**: `instances` - the set of instances whose header info is to be used **Returns:**: a set of item sets, each containing a single item **Throws:**: `java.lang.Exception` - if singletons can't be generated successfully

---


### support

```
public int support()
```

:   Outputs the support for an item set.

    :   **Returns:**: the support

---


### toString

```
public java.lang.String toString(Instances instances)
```

:   Returns the contents of an item set as a string.

    :   **Parameters:**: `instances` - contains the relevant header information **Returns:**: string describing the item set

---


### upDateCounter

```
public void upDateCounter(Instance instance)
```

:   Updates counter of item set with respect to given transaction.

    :   **Parameters:**: `instance` - the instance to be used for ubdating the counter

---


### upDateCounters

```
public static void upDateCounters(FastVector itemSets,
                                  Instances instances)
```

:   Updates counters for a set of item sets and a set of instances.

    :   **Parameters:**: `itemSets` - the set of item sets which are to be updated: `instances` - the instances to be used for updating the counters

---


### counter

```
public int counter()
```

:   Gets the counter

    :   **Returns:**: the counter

---


### items

```
public int[] items()
```

:   Gest the item set as an int array

    :   **Returns:**: int array represneting an item set

---


### itemAt

```
public int itemAt(int k)
```

:   Gest the index of the value of the specified attribute

    :   **Parameters:**: `k` - the attribute index **Returns:**: the index of the attribute value

---


### setCounter

```
public void setCounter(int count)
```

:   Sets the counter

    :   **Parameters:**: `count` - the counter

---


### setItem

```
public void setItem(int[] items)
```

:   Sets an item sets

    :   **Parameters:**: `items` - an int array representing an item set

---


### setItemAt

```
public void setItemAt(int value,
                      int k)
```

:   Sets the index of an attribute value

    :   **Parameters:**: `value` - the inex of the attribute value: `k` - the index of the attribute

---


### getRevision

```
public java.lang.String getRevision()
```

:   Returns the revision string.

    :   **Specified by:**: `getRevision` in interface `RevisionHandler`
    :   **Returns:**: the revision


---


|  |  |  |
| --- | --- | --- |
| **Overview** \| **Package** \| **Class** \| **Tree** \| **Deprecated** \| **Index** \| **Help** \| **Weka's home** | |  |
| **PREV CLASS**   **NEXT CLASS** | **FRAMES**    **NO FRAMES**     **All Classes** |  |
| SUMMARY: NESTED \| FIELD \| CONSTR \| METHOD | DETAIL: FIELD \| CONSTR \| METHOD |  |


---
